# Anthropogenetic Variability in the Group of Individuals with Febrile Seizures: Population-Genetic Study

**DOI:** 10.1155/2018/7845904

**Published:** 2018-07-05

**Authors:** Sanja Dimitrijevic, Suzana Cvjeticanin, Aleksandra Pusica, Biljana Jekic, Tamara Filipovic, Dimitrije Nikolic

**Affiliations:** ^1^Special Hospital for Cerebral Palsy and Developmental Neurology, School of Medicine, University of Belgrade, Belgrade, Serbia; ^2^Institute of Human Genetics, School of Medicine, University of Belgrade, Belgrade, Serbia; ^3^Institute of Oncology and Radiology, Belgrade, Serbia; ^4^Institute for Rehabilitation, School of Medicine, University of Belgrade, Belgrade, Serbia; ^5^University Children's Hospital Tiršova, School of Medicine, University of Belgrade, Belgrade, Serbia

## Abstract

Febrile seizures (FS) are the most common neurological disorder in childhood and are a great stress for parents due to their dramatic clinical appearance. Using test for determination of homozygously recessive characteristics in humans (HRC test) we analyzed presence, distribution, and individual combination of 20 selected genetically controlled morphophysiological traits among FS patients (N=121) and control (N=121) to determine a possible deviation in the homozygosity level and genetic loads in the group of affected children and whether there is a predisposition to the occurrence of FS. The results of our study show a statistically significant difference in the mean values of the HRC tested (${\bar{x}}_{HRC/20}$ CN = 3.2 ± 0.2; ${\bar{x}}_{HRC/20}$ FS = 4.6 ± 0.2, t= 5.74 , p< 0.0001), as well as in the distribution and variability of two studied samples (V_C_=55,3%, V_FS_= 39,6%), which indicates a complex polygenic difference among the tested groups of subjects. The differences in the degree of genetic homozygosity and variability are also present between the genders (t Cf/FSf = 4.12; t Cm/FSm = 3.98; p <0.0001) (V_Cf_=56.9%, V_FSf_= 39.3%; V_Cm_=54.1%, V_FSm_=40.1%). Obtained results indicate the enlargement of recessively homozygous genetic loads in the group of children with FS which may represent some kind of predisposition for expressivity of this type of seizures.

## 1. Introduction

Febrile seizures (FS) are one of the most common neurological disorders in children and infants. It is estimated that 2-5% of children younger than 5 years of age experience at least one epileptic seizure during the febrile period [1, 2].

FS, as defined by the American Academy of Pediatrics (AAP), is “seizure occurring in febrile children between the ages of 6 and 60 months who do not have an intracranial infection, metabolic disturbance, or history of afebrile seizures” [3].

The diagnosis of FS is based on physical examination and anamnesis taken from the guardian, aiming primarily to detect the real cause that led to a FS [4, 5].

The etiology of FS is complex and it is still the subject of numerous studies and research done in the field. However, there is strong evidence showing that heterogeneous genetic predisposition interacting with various risk factors can lead to a FS [6, 7].

There are several risk factors mentioned in literature which can cause the first FS. One of the most important factors is positive family history of FS (especially among the closest relatives) [8]. Other possible factors include high body temperature (higher level of body temperature increases the risk of a seizure occurrence) [9], preexisting neurodevelopment delay [10], and neonatal care longer than 28 days [11].

The development of epilepsy after FS is higher than in healthy population and ranges from 5.5% to 10% [12–15]. Around 13% of epilepsy patients have experienced FS once [10]. Prolonged FS can lead to mesial temporal sclerosis and to temporal lobe epilepsy, but the level of risk is still uncertain [16].

The research shows that abnormal neurological development before the FS and the occurrences of a febrile seizure among relatives, as well as the CFS, represent risk factors for emergence of epilepsy after the FS [10].

Since FS are genetically controlled, it is presumed that increased homozygosity and decreased variability in patients can be in correlation with the expression of simplex febrile seizures and complex febrile seizures.

The determination of the presence of homozygous-recessive characteristics (HRC) in individuals with FS provides an insight into whether the prevalence of homozygous or heterozygous loci on different chromosomes exists. The number of homozygous-recessive traits represents one type of indicators of the homologous chromosomal homozygosity, which can vary significantly both at the individual and at the group level [17–22].

The aim of our study is to determine a possible deviation in the homozygosity level and genetic loads in the group of affected children and whether there is a predisposition to the occurrence of FS.

## 2. Material and Methods

### 2.1. Study Design and Setting

This retrospective, observational, case-control study was conducted from September 2015 to April 2017. Parents or guardians of minors, before inclusion in the study, were informed about the full study protocol, whereby their consent was obtained. Each participant in the study, or in the case of a minor his guardian, signed an informed-consent form. The Ethical Committee of Faculty of Medicine University in Belgrade R.Serbia approved our study.

### 2.2. Subjects

We retrospectively analyzed 121 patients aged 5 to 14 years diagnosed with FS that were hospitalized or received ambulatory treatment in University Children's Hospital in Belgrade. All children who had their first febrile seizure between February 2012 and November 2014 were included in the study. All the relevant data were taken from the patients' history and per anamnesis. All patients and controls included were subjected to proper history taking, through clinical, detailed neurological examination and HRC testing. During the research, we took care to protect the privacy and anonymity of the respondent's data. The research was performed in compliance with the relevant laws and institutional guidelines.

FS were classified as simplex and complex, based on published criteria.

Simplex febrile seizures (SFS) last shorter than 15 minutes and their type is tonic-clonic. Also, they did not show signs of recidivism during the first 24 hours and were diagnosed at the patients from 6^th^ month to 5^th^ year [23].

Complex febrile seizures (CFS) were diagnosed in those patients that had focal seizure or epileptic status or seizure with the body temperature lower than 38 degrees, which occurred outside of the typical age group and finally which repeated in the first 24 hours again [24].

Epilepsy was confirmed by a history of at least 2 unprovoked seizures (except single episodes of afebrile seizures and seizures occurring in the neonatal period), accompanied by specific EEG epileptiform changes. Patients with diagnostic intracranial infection and metabolic disturbance or noncompliant patients were excluded from the study.

Patients with FS were divided into following subgroups:

**(1) By the type of seizure**

 
**SFS**: group of individuals with simple FS (N=80)
 
**CFS**: group of individuals with complex FS (N=41)

**(2) By presence or absence of epileptic seizures **

 
**WFS**: group of individuals with FS and without epilepsia (N=99)
 
**EFS**: group of individuals with epilepsia and FS (N=22).

### 2.3. Control Group

The control group (CN) was sampled randomly from preschool or school dispensaries in the city of Belgrade. CN was made of 121 healthy examinees older than 5 years of age that have never had any neurological disorders in their anamnesis or personal/family history of seizures age-matched to the children in the FS group. The examinees in control group had similar sex distribution, were raised in similar environment, and had similar socioepidemiological characteristics as patients examined in our study. The FS affected children as well as the individuals from the control group were members of the same population (Serbian).

### 2.4. HRC Test

Using HRC test (test for determination of homozygously recessive characteristics in humans) we analyzed presence, distribution, and individual combination of 20 selected genetically controlled morphophysiological traits among FS patients and control group of individuals (Table 2) [17–22, 25].

Based on fact that the studied homozygous-recessive traits are obviously controlled by genes located on different human chromosomes the results of our HRC test show the degree of genetic homozygosity and variability for 20 tested loci. Moreover, the amount of recessive homozygosity established by our HRC test is practically an estimation of homozygously expressed genetic loads present in the tested groups of individuals.

The results of HRC test show a degree of genetic homozygosity as well as the level of possible genetic loads which may indicate the presence of genetic problems which further affect the capacity of normal development, with the possibility for more extreme cases of developing specific properties including increased or decreased resistance to certain types of illnesses [18, 19, 21, 22].

In order to achieve high level of objectivity during the process of data gathering, the same person was conducting all testing. Inside the group of characteristics which show high level of variability (eye and hair color, shape of scalp, hyperextensibility of wrists), only the extreme phenotypes were considered recessive (blue eyes and hair, extremely soft hair, extreme hyperextensibility of wrists, etc.). For the purpose of testing the color blindness in examinees, the pseudoisochromatic plates test was used (Ishihara's Color-Blindness Tests ) [26].

### 2.5. Statistical Analyses

Data were analysed using SPSS v17.0 (SPSS Inc., Chicago, IL, USA). Variations in the presence of such characteristics were estimated using standard statistical procedures and by comparing the means, the variances, and shapes of the distribution between the affected and healthy individuals. We used common tests to determine the differences in the mean values, scope, and type of variability (t-test, S^2^, *χ*^2^, and V, respectively).

## 3. Results

Our results show that the degree of recessive homozygosity in all groups of patients (FS, SFS, CFS, WFS, and EFS) significantly increases with respect to the control group (Table 1) (t_CN/FS_ = 5.74; t_CN/SFS_ = 4,53; t_CN/CFS_ = 4,7; t_CN/WFS_ = 5,09; t_CN/EFS_ = 4,16; p <0,0001 ), while comparison among subgroups of the affected children shows no differences (t_SFS/CFS_ = 0.96; t_EFS/WFS_ = 1.18; p> 0.05) (Table 1). Also, variability for the tested genes in the group of FS patients decreases compared to the control group of individuals (CN: V = 55.3%; FS: V = 39.6%). It is interesting that genetic homozygosity increases and variability decreases in correlation with severity of FS expression (SFS: V = 40.5%; CFS: V = 35.05%; WFS: V = 40.2%; EFS: V = 37.6%) (Table 1).

Observing the representation of individual HRCs in persons with FS in relation to the control group, as well as within the SFS/CFS and WFS/EFS subgroups, we notice that there is a difference in the representation of all 20 traits (Table 2). In the group of patients with FS compared to the control, 11 characteristics were significantly more frequent in subjects with FS, of which 7 were in the head region and 4 at the extremities (Table 2, characteristics Nos. 1, 2, 5, 7, 8, 12, 13, 16, 18, 19, and 20). By comparing the subgroup SFS versus CFS it was found that 7 characteristics statistically significantly deviate (Nos. 3, 6, 12, 17, 18, 19, and 20), four were more common with CFS (Nos. 3, 6, 12, and 17), and two were common at SFS ( Nos. 18, 20). The three HRCs were in the head region, and the remaining four were on the extremities (Table 2). It is interesting that, by comparing the subgroups of subjects with epilepsy (EFS) with respect to the subgroup WFS (Nos. 2, 5, 6, 10, 11, 12, and 13), all HRCs that significantly deviated were in the head region, of which 2 properties were more common in the WFS group (Nos. 5,13) (Table 2).

The *χ*2-test value, for all tested traits, shows the difference in the variability between control and children with FS and this difference is highly significant (Σ X^2^ cn/fs = 109.02; p <0.001), which shows that it is their genetic dispositions that differ. Comparisons among FS subgroups show also highly significant differences (Σ X^2^ sfn/cfn = 66.7; Σ X2 efn/wfn = 89.88; p <0.001) (Table 2 ) which points out to a population-genetic difference present among them.

By comparing the average HRC values between genders in the control sample as well as in the group of FS examinees, there is no statistically significant difference (Table 3) (Cm = 3.18 ± 0.22, Cf = 3.27 ± 0.24, t = 0.26, and p> 0.5) (FSm = 4.44 ± 0.23, FSf = 4.66 ± 0.24, t = 0.69, and p> 0.5). There are minor differences in the variability between genders in the tested groups of individuals (control: Vm=54.1%, Vf=56.9%; FS affected: Vm= 40.1%, Vf=39.3%). A slightly higher variability was found in the control group of girls, while in the FS group a discretely higher variability was found in boys (Vf = 39.3%; Vm = 40.1%) (Table 3). On the other hand both FS affected females and males have significantly higher degree of genetic homozygosity and decreased variability compared to female and male individuals in the control (t Cf/FSf = 4.12t Cm/FSm = 3.98; p <0.0001) (Table 3).

## 4. Discussion

In our study, 66.1% of patients had SFS, which is consistent with previous epidemiological studies where the prevalence ranges from 55.2% to 85.6% [27, 28].

The percentage of patients with epilepsy in our study is 18.18%; this data disagrees with the data from other studies, which indicate that the percentage of patients with FS who develop epilepsy ranges from 5.5% to 10% [12–15], and the percentage of FS in the group of patients with epilepsy is about 13 % [10]. This difference stems precisely from the fact that we collected the sample in a tertiary institution, where patients with epilepsy were more likely to be observed in relation to those who did not develop epilepsy and who were given the treatment needed at the primary care institution.

In the literature, it is stated that FS is more frequent in the males, which agrees with the results of our research (47.9% of women and 52.1% of men) [29]. Studies in the territory of Serbia state that there is no difference in FS disease according to gender, while other studies indicate a more frequent occurrence of FS seizures in boys [30].

Our results clearly show a different distribution in the frequency of homozygous-recessive traits in two tested groups of individuals, which points out to the existence of a population-genetic difference between them (Tables 1 and 2). As the genes for the HRC examined are found on different chromosomes, they are considered as markers not only of these chromosomes, but also of the adjacent groups of polygenes that may have an effect on the immunological potentials and predispositions for the appearance of certain diseases, including also FS [13–16]. In children with FS a significantly higher degree of recessive homozygosity is found (CN = 3.22 ± 0.16, FS = 4.55 ± 0.16, t = 5.74, and p <0.001) (Table 1).

The results of our study show that representation in the presence of individual HRCs varies; 55% (11/20) of phenotypic characteristics are more frequent in patients with FS (Table 3), which also suggests an increased recessive homozygosity for genes that determine the tested properties and could assume that certain combinations of genes affect the occurrence of FS in children. From the results of our research, it can be assumed that the significantly increased recessive homozygosity as well as the reduced variability in the tested traits in individuals with FS leads the organism to a specific state of genetic-physiological homeostasis which could represent a certain predisposition to the occurrence of febrile seizures.

These findings coincide with the research of other authors indicating that a change in the degree of individual genetic homozygosity and variability may affect the predisposition to the occurrence of certain diseases [18, 20–22].

By comparing a group of children with SFS with a group of those with CFS as well as comparing between the group of children with epilepsy with those who did not develop epilepsy, we did not find statistical significance (t EFN/WFN = 1.18; t SFN/CFN = 0.96, p> 0.05) (Table 1). This can be explained by the higher homogeneity of these groups of subjects. But when we compared the distribution of individual HRCs between given groups, we obtained a result that showed significant differences (ΣX^2^ SFN/CFN = 66.7; ΣX^2^ EFN/WFN = 111.97; p <0.001) (Table 3 ) in genetic variability for tested loci.

This suggests that different groups of genes probably affect the onset of different clinical finding of the seizure.

The difference in the degree of recessive homozygosity among the tested groups of individuals can also suggest that genes that determine the manifestation of the disease have a pleiotropic effect, which could affect other characteristics, such as the here examined HRCs.

In addition to the increased recessive homozygosity, we have the fact that the control group had the highest number of people with 3 HRCs, while in FS patients 4 HRCs were the most common. The variability of people with SFS was lower in relation to the control group, while it was higher in relation to CFS (CN = 55.3%, SFS = 40.5%, and CFS = 35.05%) (Table 1). These results point out to the fact that other factors could also affect the appearance of the type of seizure (SFS or CFS). The established increased recessive homozygosity probably leads to a change in genetic-physiological homeostasis or to an increase in genetic loads in terms of more likely onsets of FS.

By comparing mean values of recessive properties among gender (Cm = 3.18 ± 0.22; FSm = 4.44 ± 0.23, t = 3.98, p <0.0001) (Cf = 3.27 ± 0.24; FSf = 4.66 ± 0.24, t = 4.12, p <0.0001) with the FS and the CN, we observe a significant difference between the given groups, which shows that there is a clear population-genetic difference between FS affected and nonaffected boys and affected and nonaffected girls.

This indicates increased homozygosity and reduced variability of both boys and girls with FS (Table 3), with possible involvement of homo- and hemizygous X-chromosomes.

There is a limit to this study. The origin of genetic determination and type of inheritance needs to be analysed better and thus the combination of tested traits that we used in our study should be improved further. Further study on a larger sample is advised, because there is another limiting factor which relates to the number of patients.

When studying morphophysiological traits as markers of specific genes at human chromosomes, HRC test may predict some developmental potential of human individuals and as such should be applied comparatively, together with more sophisticated methods that are used in contemporary genetics and biomedicine.

## 5. Conclusion

Obtained results indicate the enlargement of recessively homozygous genetic loads in the group of children with FS which may represent some kind of predisposition for occurrence of febrile seizures.

## Figures and Tables

**Table 1:** The presence of homozygous recessive characteristics (HRCs) based on the study of 20 qualitative morphophysiological traits in the groups of individuals with different types of febrile convulsions and epilepsy.

	CN	FS	SFS	CFS	WFS	EFS
	N=121	N=121	N=80	N=41	N=99	N=22
${\bar{x}}_{hrc/20}$	3.22 ± 0.16	4.55 ± 0.16	4.39 ± 0.2	4.71 ± 0.26	4.45 ± 0.18	4.95 ± 0.4

SD	1.78	1.80	1.78	1.65	1.79	1.82

V	55,3%	39,6%	40.5%	35.03%	40.2%	37.6%

		t_CN/FS_ = 5.74*∗∗∗*	t_CN/SFS_ = 4,53*∗∗∗*		t_CN/EFS_ = 4.16*∗∗∗*	
				t_CN/CFS_ = 4.7*∗∗∗*		
t_CN/WFS_=5.09*∗∗∗*						
*∗∗∗*p<0.0001				t_SFS/CFS_ = 0.96		t_EFS/WFS_ = 1.18

**CN**: control group;** FS**: febrile seizure;** SFS**: simple febrile seizures; **CFS**: complex febrile seizures; **WFS**: febrile seizures without epilepsia; **EFS:** epilepsia.

**Table 2:** Frequencies of homozygous recessive characteristics in the groups of FS patients and control.

Homozygous recessive trait	**CN**	**FS**	**CN/FS**	**SFS**	**CFS**	**SFS/CFS**	**WFS**	**EFS**	**WFS/EFS**
	N=121	N=121	X^2^	N=80	N=41	X^2^	N=99	N=22	X^2^
1. Blond hair	7.4	14	7.11*∗∗*	12.5	17.07	1.46	13.1	18.18	1.69

2. Straight hair	30.6	43.8	6.92*∗∗*	43.75	48.78	0.55	40.4	59.1	7.28*∗∗*

3. Double hair whorl	9.9	13.2	1.33	10	19.51	6.83*∗∗*	13.1	13.63	0.02

4. Opposite hair whorl orientation	6.6	7.4	0.12	7.5	7.32	0.004	9.09	0	0

5. Soft hair	15.7	29.7	6.08*∗*	27.5	34.15	1.45	32.32	18.18	8.59*∗∗*

6. Continuous hairline	26.4	27.3	0.03	23.75	34.15	3.86*∗*	23.23	45.45	9.72*∗∗*

7. Attached ear lobe	7.4	12.4	4*∗*	11.25	14.63	0.89	12.12	13.63	0.18

8. Ear without Darwinian knot	7.4	14.9	9*∗∗*	13.75	17.07	0.74	14.14	18.18	1.02

9. Blue eyes	24.8	19	1.63	20	17.07	0.44	19.19	18.18	0.05

10. Color blindness	0.8	1.6	1	2.5	0	0	1.01	4.54	7.53*∗∗*

11. Speaking deficiency (guttural “r”)	17.3	20.7	0.76	17.5	24.39	2.33	18.18	31.81	8.03*∗∗*

12. Inability to curve the tongue	8.3	18.2	14.4*∗∗∗*	12.5	31.71	20.56*∗∗∗*	14.14	36.36	24.24*∗∗∗*

13. Inability to roll the tongue	14	35.5	20.22*∗∗∗*	36.25	34.15	1.12	38.38	22.72	8.59*∗∗*

14. Right thumb over left thumb	39.7	33.9	1.02	36.25	29.27	1.5	34.34	31.81	0.19

15. Top joint of the thumb >45°	13.2	17.3	1.56	16.25	19.51	0.59	18.18	13.63	1.33

16. Proximal thumb extensibility	21.5	33.9	8.65*∗∗*	32.5	36.58	0.48	32.32	40.9	2.04

17. Thumb backward movability	19.8	24	1.04	21.25	31.71	4.29*∗*	22.22	31.81	3.51

18. 3 strings in the wrist	24	35.5	15.04*∗∗∗*	41.25	24.39	4.95*∗*	37.37	27.27	3.23

19. Left-handedness	19.8	12.4	4.17*∗*	10	19.51	6.83*∗∗*	13.1	9.1	2.3

20. Index finger longer than the ring finger (in males; opposite in females)	26.4	37.2	5.28*∗*	43.75	24.39	8.74*∗∗*	36.36	40.9	0.53

**∑X** ^**2**^			**109.02** *∗∗∗*			**66.7** *∗∗∗*			**89.88** *∗∗∗*

*∗*p<0.05 *∗∗*p<0.01*∗∗∗*p<0.001									

**CN**: control group;** FS**: febrile seizure;** SFS**: simple febrile seizures; **CFS**: complex febrile seizures; **WFS**: febrile seizures without epilepsia; **EFS:** epilepsia.

Significant differences in frequencies of studied homozygous recessive characters.

CN/FS Nos. 1, 2, 5, 7, 8, 12, 13, 16, 18, 19, and 20.

SFS/CFS Nos. 3, 6, 12, 17, 18, 19, and 20.

EFS/WFS Nos. 2, 5, 6, 10, 11, 12, and 13.

**Table 3:** Distribution of homozygously recessive characteristics (HRC-test) based on the study of 20 qualitative morphophysiological traits among genders (groups of FS affected and control).

	Cm	Cf	FSm	FSf
	N=61	N=60	N=62	N=59
${\bar{x}}_{hrc/20}$	3.18 ± 0.22	3.27 ± 0.24	4.44 ± 0.23	4.66 ± 0.24

SD	1.72	1.86	1.78	1.83

V	54.1%	56.9%	40.1%	39.3%

	t_Cm/CNf_ = 0.26		t_FSm/FSf_ = 0.69	
	t_Cf/FSf_ = 4.12*∗∗∗*		t_Cm/FSm_ = 3.98*∗∗∗*	
*∗∗∗*p<0.0001				

**Cm**: control group, male;** FSm**: febrile seizure, male; **Cf**: control group, female; **FSf**: febrile seizure, female.

## Data Availability

The data used to support the findings of this study are included within the article.
